# Pandemic Influenza Outbreak on a Troop Ship—Diary of a Soldier in 1918

**DOI:** 10.3201/eid1811.AD1811

**Published:** 2012-11

**Authors:** Jennifer A. Summers

**Affiliations:** University of Otago, Wellington, Department of Public Health, Wellington South, New Zealand

**Keywords:** influenza, pandemic, epidemiology, historical, military, World War I, New Zealand

## Abstract

A newly identified diary from a soldier in 1918 describes aspects of a troop ship outbreak of pandemic influenza. This diary is the only known document that describes this outbreak and provides information not officially documented concerning possible risk factors such as overcrowding and the suboptimal outbreak response by military leaders. It also presents an independent personal perspective of this overwhelming experience.

A previous study described the epidemiology and risk factors for death from pandemic influenza in 1918 aboard a World War I (WWI) New Zealand troop ship (*1*). This outbreak aboard His Majesty’s New Zealand Transport (HMNZT) Tahiti represents a worst-case scenario for a novel infectious disease outbreak, occurring in a crowded setting with limited medical resources. Although this ship outbreak of pandemic influenza was not the most severe reported during 1918 (*2*,*3*), it was a notable outbreak that had a cumulative mortality rate of 68.9 cases/1,000 population and estimated cumulative morbidity rate of 90%.

A newly identified diary from a soldier on this ship has recently been transcribed by the author’s grandson for July 10, 1918, through January 31, 1919 (Figure) (J Hansen, unpub. data). The diary accounts were not part of the official inquiry made into this outbreak or subsequent report (*4*,*5*). Therefore, this diary, the only known document of its nature, is an independent and unofficial account of this outbreak. The author of the diary, now deceased, was a rifleman in the 40th Reinforcements of the New Zealand Expeditionary Force aboard HMNZT Tahiti. Before the war, he was a telegraphist, and later served in the New Zealand Air Force during World War II (*6*).

**Figure:**
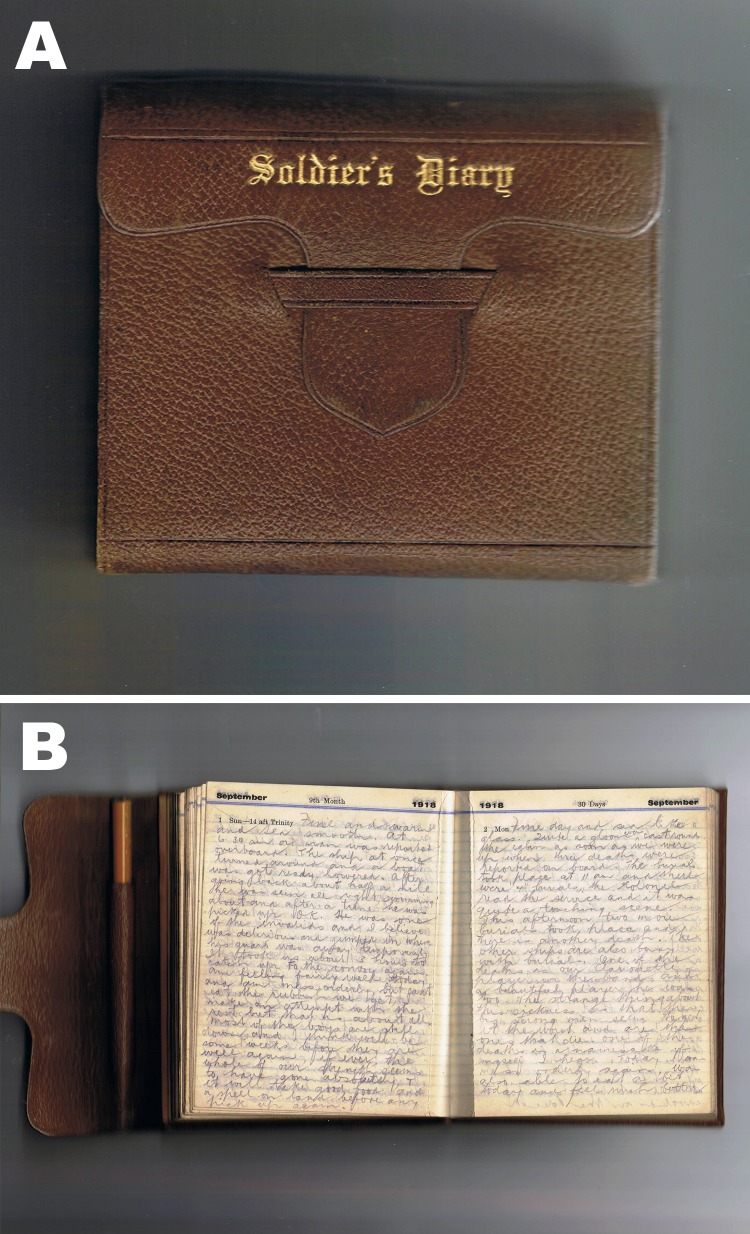
A) Front cover and B) entry of a diary from a soldier on board His Majesty’s New Zealand Transport Tahiti during a period of pandemic influenza, July 10, 1918, through January 31, 1919.

HMNZT Tahiti left New Zealand on July 10, 1918, and, after stops in Australia and South Africa, joined a military convoy in Freetown, Sierra Leone, in western Africa. This voyage was among the last to supply new New Zealand reinforcements to Europe during WWI.

10 July (Diary entry, day of embarkment, Wellington, New Zealand):

We left the wharf at 1.55 pm and finally set sail at 5 pm. Started off in fine weather and all well. There were 4 companies of infantry, A B C & E, and one coy of artillery and our own coy, the 40th Specs on board in this trip & no doubt the total number will be about 1200, truly a large number to be packed on this vessel, which I hear is about 7000 tonnes, and where I am domiciled, we are packed in like sardines in the bows of the ship ‘tween decks. Our bunks consist of hammocks strung to the ceiling and we are packed right across the ship longways & sideways & every inch of space is utilised, for underneath are our mess tables which are also our writing tables etc, & we have to live down here in rough weather. The food is very good and plenty of it. We have a band aboard & I joined same. Played some tunes before we finally sailed.

The language in the diary suggests that the respondent was a fairly thoughtful observer and not prone to exaggeration, because his diary entries accurately describe, when compared with the official report, ship hospital admission numbers and details aboard during the outbreak. For example, the first cases of influenza (n = 35) were reported in the diary on August 26: *“*… the hospital is over full and also a dozen patients on deck.” This is consistent with the official report, which documents that when “… the number of cases exceeded the capacity of the Hospital (36) more and more deck space was utilised for Hospital purposes.”

The soldier himself contracted influenza during the outbreak, but his illness was not incapacitating, and he maintained daily entries into his diary throughout the outbreak.

27 August (diary entry; 1 day after setting sail from Freetown, Sierra Leone):

Fairly warm and showers of rain occasionally. An epidemic of influenza seems to have broken out on board. Sixty odd on this morning’s sick parade and 24 admissions to the hospital. Have caught it myself of course but so far am able to get about & think I will hang out all right but feel very crook. Saw a few porpoises quite near the boat today. We are still travelling in the same formation but the distances from each boat varies. No band this morning, as a lot of the members are not well. This afternoon we pasted some music on to cards instead of practicing. This morning we had a lecture for half an hour on transport etc in England and was a most idiotic lecture too. Had no object in it that I could see.

2 September (diary entry; part of military convoy off the coast of Western Africa):

Fine day and sea like glass. Quite a gloom was cast round the cabin as soon as we were up when three deaths were reported on board. The burials took place at 11am and there were 4 burials. The Colonel read the service and it was quite a touching scene. This afternoon two more burials took place and there is another death. The other ships are also busy with burials. One of the deaths is our Clarionette player in the band and a beautiful player he was too. The strange thing about this sickness is that the big strong men seem to get it the worst and are the ones that die. One of the deaths is namesake of myself I hear. Today was mess orderly again. Was also able to eat a bit today and feel much better.

10 September (diary entry; arriving in England):

Fine but very cold & sea fair. Land was in sight this morning and all very excited in the thought of getting on land again for good.… The country here looks really beautiful green fields and ploughed land with forests here and there. … One man died as we were coming into port.

Despite the obvious ongoing outbreak, officials continued with war preparation, regardless of the potential transmission risk. The diary states that after the outbreak and situated in England, the troops were required to parade, although they were weak: “most of us are not too strong yet and got a bit fagged.” However, later treatment of the troops does imply that military officials were not lacking empathy, because a brigadier general promised the troops “that when all danger of infection was over we would be granted a week’s leave and free rail pass anywhere, so we are on a win if all goes well.” There is no reference made in the inquiry regarding this leave; however, the soldier documents on October 10: “Tomorrow we are to get our weeks leave which we have been looking forward to for so long.” Also, the following week’s diary entries describe travel to Edinburgh and Glasgow: “Quite a treat to get in a decent bed again and makes one feel almost homesick. Hurray, won’t need to be up with the blasted bugle in the morning.”

A crowded environment is an acknowledged risk factor for influenza transmission and probably played a role in this particular outbreak (*1*). Furthermore, the official inquiry concluded that the cause of the high death rate on the ship was caused by “the virulent nature of the infection, which affected a large number of men massed together on a ship where the ventilation was defective owing to the enforced closing of the ports and the absence of any form of recognized artificial ventilation.” (*4*) The diary makes several references to the disagreeable conditions aboard. On July 10, (pre-outbreak), the soldier writes, “we are packed in like sardines,” and “we are packed right across the ship longways & sideways,” on August 29 (mid-outbreak), “men are packed in such a small space.” On September 5 (mid-outbreak), he elaborates, “more deaths and burials total now 42. A crying shame but it is only to be expected when human beings are herded together the way they have been on this boat.” The inquiry concluded that the crowding aboard ship was justified given the necessities of war and that it was no more excessive than on other troop ships during this period.

It was clear at the time of the inquiry that medical provision on board the ship was inadequate for coping with an outbreak of this scale. The inquiry and report documented that the nursing staff were “overwhelmed with work,” criticized the work of the medical orderlies, and indicated that there was a lack of medical supplies (*4*,*5*). The August 31 entry in the diary supported this: “the ship has also run short of medicine now, this sickness must have been something they did not bargain for.” However, as the previous study concluded (*1*), medication for influenza during 1918 was unlikely to have had an effect on the risk for death. Referring to the overwhelmed medical staff, the diary corroborates the findings of the inquiry: “two or three hundred more on sick parade today and the nurses and doctors have their hands full all right now.”

Food supplies on board were discussed in the diary and the inquiry, with some conflicting entries. A pre-outbreak diary entry stated: “The food we are having is very good & plenty of it so far.” However, even after the ship was stocked up with provisions in Freetown, Sierra Leone, a later entry (mid-outbreak) noted: “There are tons of meat etc wasted just for the want of cooking into a decent dish… but the muck we get to eat does not tempt any one to eat.” This statement suggests that the overall food quality during the outbreak may have been poor. This account is partially consistent with the inquiry findings, which acknowledged some problems with food quality. However, the inquiry does state: “it is apparent from the evidence that there was an abundance of other foods to supplement the bread and meat on the occasions on which they were not satisfactory” (*4*). However, it is not clear from either source whether food provision was the problem or whether there was a failure to organize able military staff to cook food (assuming the ship’s original cooks were unwell).

Regarding unsatisfactory food, a diary entry made at mid-outbreak noted: “Heard today that the Sergeant’s mess have received from the skipper a certificate asking them to sign to effect that the food and general comfort on this ship was all that could be desired and not one would sign it.” This entry suggests that military leaders aboard the Tahiti were aware that the overcrowding situation existed and that the outbreak response was lacking (as was to some extent confirmed by the subsequent inquiry).

The last influenza-related death among the 40th Reinforcements occurred in England on October 3, 1918, at the beginning stages of the second wave of pandemic influenza (*7*). The October 24 diary entry states: “Very foggy and bitterly cold first thing in the morning. Band parades as usual. Learnt how to counter march this morning.… We were all inoculated this morning for the purpose of counteracting the influenza plague. I believe there is an epidemic of it in camp at present.”

This diary provides an insightful and relevant snapshot of one soldier’s experience during an influenza outbreak. The chronological account in this soldier’s diary of this troop ship outbreak of pandemic influenza also supports issues raised in other historical documents: the potential for crowded quarters to amplify influenza transmission and the inadequate provision of medical resources (both supplies and staff), which were also identified in the official inquiry. However, new issues are raised by the information noted in this diary: the likelihood for poor organization of the provision of food, interference in official accounts of conditions aboard the ship, and military rigidity in the wake of an overwhelming influenza outbreak. Release of this account enforces the idea that historical data sources should be routinely sought by researchers examining the complex issues related to the epidemiology and control of past pandemics.

None of the 40th Reinforcements soldiers experienced combat as part of WWI; the war ended on November 11, 1918. However, of the 1,117 members of the 40th Reinforcements on board, >90% were sickened by the pandemic influenza strain, and 77 died. This caused one of the highest mortality rates from any cause among New Zealand military units. The diarist refers to the outbreak as “quite a catastrophe,” and it was an experience not likely to have been forgotten by those aboard this ill-fated voyage of HMNZT Tahiti.
